# Making a decision about trial participation: the feasibility of measuring deliberation during the informed consent process for clinical trials

**DOI:** 10.1186/1745-6215-15-307

**Published:** 2014-07-30

**Authors:** Katie Gillies, Glyn Elwyn, Jonathan Cook

**Affiliations:** Health Services Research Unit, University of Aberdeen, 3rd Floor, Health Sciences Building, Foresterhill, Aberdeen AB25 2ZD UK; The Dartmouth Center for Health Care Delivery Science, Dartmouth College, 37 Dewey Field Road, Hanover, NH 03755 USA; Nuffield Department of Orthopaedics, Rheumatology and Musculoskeletal Sciences, University of Oxford, Nuffield Orthopaedic Centre, Windmill Road, Oxford, OX3 7HE UK

**Keywords:** Clinical trial, Decision-making, Informed consent, Measure

## Abstract

**Background:**

Informed consent of trial participants is both an ethical and a legal requirement. When facing a decision about trial participation, potential participants are provided with information about the trial and have the opportunity to have any questions answered before their degree of ‘informed-ness’ is assessed, usually subjectively, and before they are asked to sign a consent form. Currently, standardised methods for assessing informed consent have tended to be focused on aspects of understanding and associated outcomes, rather than on the process of consent and the steps associated with decision-making.

**Methods:**

Potential trial participants who were approached regarding participation in one of three randomised controlled trials were asked to complete a short questionnaire to measure their deliberation about trial participation. A total of 136 participants completed the 10-item questionnaire (DelibeRATE) before they made an explicit decision about trial participation (defined as signing the clinical trial consent form). Overall DelibeRATE scores were compared and investigated for differences between trial consenters and refusers.

**Results:**

No differences in overall DelibeRATE scores were identified. In addition, there was no significant difference between overall score and the decision to participate, or not, in the parent trial.

**Conclusions:**

To our knowledge, this is the first study to prospectively measure the deliberation stage of the informed consent decision-making process of potential trial participants across different conditions and clinical areas. Although there were no differences detected in overall scores or scores of trial consenters and refusers, we did identify some interesting findings. These findings should be taken into consideration by those designing trials and others interested in developing and implementing measures of potential trial participants decision making during the informed consent process for research.

**Trial registration:**

International Standard Randomised Controlled Trial Number (ISRCTN) Register ISRCTN60695184 (date of registration: 13 May 2009), ISRCTN80061723 (date of registration: 8 March 2010), ISRCTN69423238 (date of registration: 18 November 2010)

## Background

Informed consent for research participation is an ethical and legal requirement that covers aspects such as capacity, disclosure, understanding, voluntariness and permission [1]. There is a regulatory requirement to provide information about these key features to potential clinical trial participants and to assess, usually subjectively, their understanding of said information and recognition that participation is optional before their consent is obtained [2, 3]. Current guidelines that regulate informed consent for clinical trials tend to focus more on the information provided to potential trial participants than on the understanding and quality of the decision that these individuals reach [3]. The subjective assessment of informed consent for clinical trials, and the potential difficulties associated with it, has led several studies to develop objective measures of informed consent for clinical trials [4, 5]. These objective measures of informed consent are often specific to a particular population or clinical condition and largely focus on understanding of (some or all of) the key elements of informed consent, namely: capacity, disclosure, understanding, voluntariness and permission [6–10]. Many of the developed tools are study-specific, but some validated measures exist. Probably the most widely used validated measure is the Quality of Informed Consent (QuIC) tool, which was developed in cancer clinical trials [6]. The QuIC measures aspects of both objective comprehension (for example, understanding the trial) and subjective understanding (for example, ‘informed-ness’). More recent validated measures of informed consent for clinical trials are focused on particular aspects of informed consent, such as randomisation and placebo [11] and the therapeutic misconception [12].

Some of the objective measures of informed consent for clinical trials tend to conceptualise and operationalise aspects of understanding in relation to clinical trials in a somewhat limited way. Several focus on knowledge of the trial in a general sense, and others check a participant’s ability to correctly recall information without considering comprehension; but all lack consideration of other aspects that may be important to the decision-making process (for example, preference construction, affective forecasting and determining what matters most to an individual) [6–12]. As such, the current evidence base on informed consent measures highlights a focus on knowledge and understanding, which, though important building blocks, are not the only components required for decision-making. There also remains a lack of recognition of the importance of the ‘process’ in many of these measures, evidenced by a focus on outcomes rather than on the steps leading up to such outcomes [6–12]. This is of particular relevance when considering that informed consent is defined as ‘a process by which a subject voluntarily confirms his or her willingness to participate in a particular trial, after having been informed of all aspects of the trial that are relevant to the subject’s decision to participate’ [3]. Existing measures of informed consent tend to make implicit assumptions about the type of information that is relevant to a potential participant’s decision-making and based largely on the information or concepts prespecified in the guidance [6–12]. Moreover, most of the interventions to date that aim to improve informed consent for randomised controlled trials are focused on improving understanding [4, 5]. Evaluation of these interventions has not led to identification of an optimal method, and, as such, it may be important to consider new interventions focused on the decision-making process and measures relevant for their assessment [13].

The majority of studies in which researchers have assessed informed consent for clinical trials have involved implementation of tools to measure outcomes after an explicit decision was made (that is, after consent was given or not) [4, 5]. Measuring aspects of decision-making after a decision has been made may be susceptible to bias due to the influence of outcomes attributed to the decision [14]. For example, a decision about trial entry might be viewed more positively if participants receive (or, in a blinded trial, believe they have received) the intervention for which they had a preference and vice versa. It has been suggested that the decision-making process for treatment choices can be separated into the categories of *deliberation about processes* and *determination of decisions* and that these steps require different evaluation approaches [14]. A potential advantage of this is that measuring deliberation could provide a measure of the decision-making process without its being biased by any influences derived from decision-making outcomes. The deliberation step of decision-making has been proposed to have several overlapping stages, ranging from information-seeking, information-processing and assessing knowledge sufficiency to imagining counterfactuals, emotional processing, affective forecasting, preference construction and readiness to make a choice [14]. These steps come together to inform and determine the decision stage [14]. Some of the stages of deliberation involve aspects that are also traditionally associated with aspects of informed consent, specifically seeking information, processing information and assessing knowledge sufficiency and imagining pros and cons. Individuals’ consideration of whether to provide informed consent for clinical trials as a process can be broken down into two stages: deliberation (trial is introduced, patient information leaflet is provided and a discussion about participation is conducted) and determination (decision to participate or not as demonstrated by the signing of a consent form). There is some evidence in the literature that, during explicit deliberation about preferences, potential trial participants are more likely to become more uncertain and amenable to trial participation [15]. This finding supports the notion that measuring deliberation may provide data that is not biased by decisional outcomes, but illustrative of the overall consent process [15]. It also supports a hypothesis that deliberation during the informed consent process may differ between trial consenters and refusers. For example, consenters may feel more ready to make a decision and correspondingly may assess their state of deliberation as higher compared to refusers, or vice versa. In further support of this hypothesis, studies have shown a potential difference between trial consenters and refusers on specific decision-making outcomes. Satisfaction (relating to the decision) has been suggested to be higher in trial consenters than in refusers, and decisional conflict (a measure of uncertainty) has been shown to be lower amongst consenters [16, 17].

Rather than being focused solely on the exact information that is provided in the trial information leaflet and using informed consent measures as an assessment of recall, which has tended to be the focus to date, a new approach to assessing the decision-making process in clinical trials could be advantageous. Specifically, by evaluating more general aspects of the decision and the associated process, which are operationalised in a way that focuses on the individual. A further benefit of a more general decision-making measure for clinical trial participation could be realised by developing a measure that is amenable to different trial contexts, thus providing a way of improving comparisons across trials and increasing the generalisability of findings in individual studies and meta-analyses.

To date, to our knowledge, there have been no studies in which researchers have developed a tool to measure informed consent to participate in clinical trials that encompasses aspects of the decision-making process beyond ‘understanding’ and that are aimed at measuring the process before an explicit decision is made. In this study, we report the use of a tool (DelibeRATE) to measure the deliberation stage of the decision-making process in the context of decisions about trial participation. Specifically, we investigated potential trial participants’ deliberation when making a decision about trial participation and whether this deliberation differs between trial consenters and refusers.

## Methods

### Participants

This study is nested within three clinical trials that were actively recruiting patients at the time it was carried out. Appropriate trials were purposively selected from among the Centre for Healthcare Randomised Trials (CHaRT) portfolio of ongoing clinical trials. CHaRT is a registered clinical trials unit of the UK Clinical Research Collaboration which specialises in pragmatic trials of complex interventions. The chief investigators of the identified clinical trials were approached, and their agreement was sought prior to study commencement.

Potential trial participants were identified from among individuals approached in each of the actively recruiting clinical trials. Two of the trials were surgical trials, one in gynaecology recruiting postmenopausal women (ISRCTN 60695184 (date of registration: 13 May 2009) and the other in general surgery recruiting from a broad population (ISRCTN 8006172 (date of registration: 8 March 2010)). The third trial was a placebo-controlled drug trial set within urology, also recruiting from a broad population (ISRCTN 69423238 (date of registration: 18 November 2010). More details on each of the included trials can be found in Table 1. To minimise burden for individual recruiters, at least three centres per trial were asked to participate, each providing 20 completed questionnaires per site to give a target total of 180 completed questionnaires for analysis. At the time we developed this study, no published studies in which the DelibeRATE tool was used were available to inform the sample size. The informal sample size calculation was based upon obtaining 200 recruits overall from 5 recruiting trials with 10 recruits per centre and 40 per trial. Due to the administrative burden, recruitment goals were not achieved at two of the five sites.

**Table 1:** **Characteristics of trials included in the DelibeRATE study** ^**a**^

Trial characteristics	Trial 1	Trial 2	Trial 3
Clinical condition	Vaginal prolapse	Haemorrhoids	Ureteric stones
Trial design	RCT and comprehensive cohort	Simple parallel design	Simple parallel design
Sample size, *N* (*n*)	4,500 (2,250 randomised)	800	1,200
Recruitment rate (%)	49	74	56
Arms	3 in each repair arm (4 different interventions)	2	3
Intervention	Surgery (RCT split by primary or secondary repair	Surgery	Drug
	*Primary repair randomisation*	1. Traditional excisional haemorrhoidectomy	1. Calcium channel blocker
	1. Standard repair	2. Stapled haemorrhoidopexy	2. α blocker
	2. Standard repair with biological mesh		3. Placebo
	3. Standard repair with nonabsorbable mesh		
	*Secondary repair randomisation*		
	1. Standard repair		
	2. Standard repair with nonabsorbable mesh		
	3. New repair with mesh kit		
Blinding	Participants and outcome assessors (for patient-reported outcomes)	Participants and outcome assessors (for patient-reported outcomes)	Participants, caregivers and outcome assessors
Number of sites	15	31	24
Primary outcome (clinical or patient-reported and timing)	Patient-reported at 2 years postrandomisation	Patient-reported at 2 years postrandomisation	Clinical at 4 weeks and patient-reported at 12 weeks postrandomisation
Parent trial participant characteristics			
Median age (IQR)	61 (52 to 68)	49 (20 to 40)	44 (34 to 52)
Sex (% females)	100	48	19

^a^RCT, Randomised controlled trial.

### DelibeRATE tool development

The development of the DelibeRATE tool was not part of this study and has been reported elsewhere [14, 18]. In brief, items included in the original DelibeRATE tool were based on the conceptualisation of the decision-making process as one of deliberation (which includes a predecisional process and an act of decision determination) rather than on the outcome of the decision [14]. Through iterative group work, items that mapped onto the concept of deliberation were developed and operationalised (that is, recognising that decisions are needed, identifying options, describing options and deliberating about the attributes) prior to making judgements and comparisons and constructing preferences. The scale was used in a study in which researchers evaluated the impact of a web-based decision-making aid for breast cancer treatment options [18]. This previous study showed the items in the tool to have good internal reliability, with Cronbach’s α values of 0.945 (preintervention) and 0.960 (postintervention) [18]. The previously published tool had undergone further refinement by the development team before being used in the study reported in this article. Specifically, previously each item in the published tool was rated on a seven-point scale anchored by ‘strongly agree’ and ‘strongly disagree’ [18]. In the updated version of the tool that was provided, and used, for this project, the responses for each item had been revised to ‘yes’, ‘no’ and ‘unsure’, which was a pragmatic decision by the developers (from [18]) to make it easier to score. Also, the previously published tool was composed of nine items. However, the tool provided, and used in this study, contained ten items, with one of them (item D3 from [18]) split into two separate items—advantages (item 3) and disadvantages (item 4)—rather than being combined.

**Figure 1:**
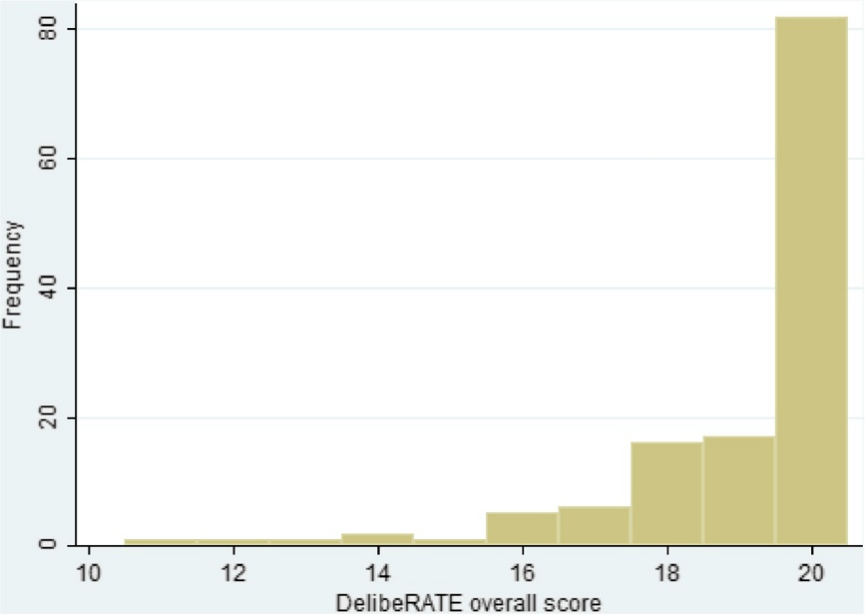
**Distribution of overall DelibeRATE scores.** DelibeRATE scores ranged from 0 to 20. No participants scored less than 11.

The DelibeRATE tool was originally developed to measure decisions about treatment [18]. As such, the wording was amended (by KG and JC) to ensure that items were explicit about the decision being related to trial participation. As such, all of the items in the tool were adapted to incorporate the phrase ‘of participating in the trial or not’. The modified DelibeRATE tool is shown in Figure 1 No validation of the scale was conducted in the context of trial participation decisions.

### Data collection

**Figure 2:**
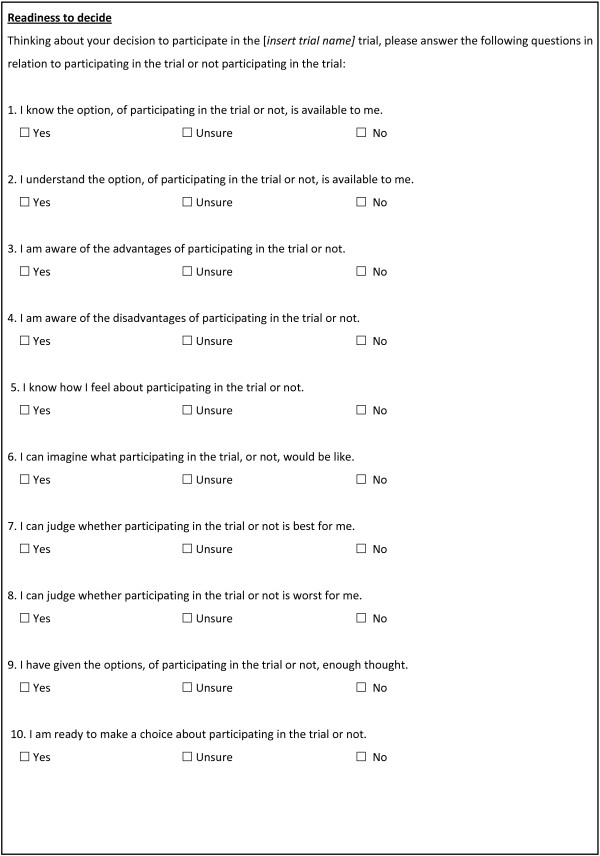
**The DelibeRATE tool.**

Following introduction of the parent trial by the recruiter, as per individual trial protocols, each patient was given the DelibeRATE questionnaire. The questionnaire included a short description of the study and instructions for completion. Once the patients completed the questionnaire, they either returned it sealed in an envelope to the recruiter for posting to the study office or, if they completed it at home, returned it to the study office in the post, depending on the nature of the recruitment process in each individual trial. The study questionnaire was composed of a short demographic section and the DelibeRATE tool. The DelibeRATE tool is a short, ten-item tool which requires each question to be rated on a three-level Likert scale and takes participants approximately 5 minutes to complete (Figure 2). The recruiters were asked to ensure that the questionnaire was completed before the decision to participate in the parent trial was made, which, for the purposes of this study, occurred before explicit written consent to participate in the parent trial was recorded. In addition to completing the DelibeRATE questionnaire, participants were asked to answer questions about their age, sex, educational attainment and previous trial participation.

The recruiter was asked to complete a short data form about each potential participant. The following data were recorded on the form: who provided the potential participant with the patient information leaflet for the trial, who had the consent discussion with the potential participant, how long the potential participant had to decide about taking part in the trial and whether the patient consented to participate in the clinical trial for which they were initially approached.

### Analysis

Participant and trial characteristics are presented using summary statistics as appropriate. The maximum DelibeRATE questionnaire overall score was 20, with each item being scored from 0 to 2 (0 = No, 1 = Unsure, 2 = Yes). The overall DelibeRATE score and individual question scores were analysed and are presented using summary statistics such as the median and interquartile ranges or number per category and percentage as appropriate. The higher the score, the readier potential participants were to making a decision about trial entry. A generalised estimating equation approach was planned to analyse the DelibeRATE score, but it was not carried out because the participating recruiting centres and recruiters were fewer than anticipated. Instead, to determine whether there was a difference in DelibeRATE scores between participants consenting or not, we performed a Mann–Whitney *U* test (two-sided, 5% significance level). Similarly, differences in the time taken to decide were tested using a Mann–Whitney *U* test or Kruskal-Wallis test as appropriate. Correlations were calculated using Spearman’s correlation statistic.

The study was approved by the North of Scotland Research Ethics Committee 1 (11/AL/0271). As the study was multicentred, the original ethics application, as approved, included appropriate review of the research with respect to recruitment of participants across all included sites. NHS Research and Development approval was sought from individual committees for each of the sites and included NHS Ayrshire & Arran, NHS Grampian, NHS Highland, Central Manchester University Hospitals NHS Foundation Trust, County Durham and Darlington NHS Foundation Trust, Plymouth Hospitals NHS Trust and South Devon Healthcare NHS Foundation Trust. Participants were not asked to complete an additional consent form for this study. Instead, consent was implied by completion and return of the questionnaire as approved by the Research Ethics Committee (see above).

## Results

### Participant characteristics

A total of 136 potential trial participants completed the DelibeRATE questionnaire between September 2011 and June 2013. Table 2 provides details of the demographic characteristics of the sample of participants who completed and returned the questionnaire. They represented three clinical trials and seven individual sites, with five sites recruiting to trial 1, four sites recruiting to trial 2 and three sites recruiting to trial 3 (Table 2). There were more females (75%) than males (25%) in the respondent sample, which was due to one of the trials (trial 1) being set within gynaecology and to that trial contributing significantly more participants (60%) than either of the other two. The median age of the participants, 55 years, also reflected the specific context of trial 1 because it is a trial set within a condition in postmenopausal women. Participants’ ages across the sample ranged from 19 to 81 years. The participants had varied levels of education, with 28% having no formal education. In relation to demographics concerning trial characteristics, only a small number (12%) of the participants had previously taken part in a clinical trial. Research nurses were most often the person providing the patient with the trial information leaflet (82%) and the person responsible for taking consent for the clinical trial (81%). The majority (94%) of the participants who returned and completed the DelibeRATE questionnaire consented to participate in the trial. The time taken to decide about participating in the trial ranged from 30 minutes to 24 weeks, with the median time being 24 hours.

**Table 2:** **Participant demographics**

Demographic characteristics	***n***(%)
Number of participants by trial	
1	81 (60)
2	27 (20)
3	28 (20)
Number of participants by site (recruiting to trial 1, 2 or 3)	
1 (trial 1, 2 and 3)	46 (34)
2 (trial 2 and 3)	14 (10)
3 (trial 3)	8 (6)
4 (trial 2)	6 (4)
5 (trial 1)	18 (13)
6 (trial 1 and 2)	25 (18)
7 (trial 1 and 2)	19 (14)
Median age, yr (IQR)	55 (43 to 65)
Males	34 (25)
Educational attainment^a^	
No formal education	38 (28)
Secondary	59 (44)
Higher	38 (28)
Previous trial participation (number of trials)	
0	120 (88)
1	13 (10)
2	2 (2)
3	1 (1)
Person providing patient info leaflet	
Research nurse	111 (82)
Consultant	8 (6)
Specialist Registrar	1 (1)
Other	16 (12)
Person taking consent	
Research nurse	109 (81)
Consultant	7 (5)
Specialist registrar	2 (1)
Other	17 (13)
Consented to trial	128 (94)
Median time to make decision, days (IQR)	1 (0.04 to 14)

^a^Data for one respondent are missing.

### DelibeRATE scores

There was no significant difference in the overall DelibeRATE scores, with a median of 20 (IQR = 18 to 20). The overall score ranged from 11 to 20, with only 5% of participants scoring 15 or below (Table 3 and Figure 2). Similarly, there was no significant difference in scores for individual items in the DelibeRATE tool. However, some items did exhibit more variability in respondents answering ‘unsure’ and ‘no’ than others. For example, though 84% of respondents scored item 4 as ‘Yes’, 12% as ‘Unsure’ and 4% as ‘No’, items 6, 7 and 8 had similar frequency distributions (Table 4). There was no significant difference (median = 20 (IQR = 18 to 20) versus median = 20 (IQR = 11 to 20); *P* = 0.669) between overall scores for those who decided to participate versus those who did not. There was evidence of a weak correlation of age (Spearman’s correlation = −0.19; *P* = 0.29) to overall score (Table 5), but not of a difference in the overall score to other factors potentially predictive of poor understanding (for example, educational attainment (*P* = 0.528)) or a difference in previous trial participation (*P* = 0.859). Time to decide (Table 5) did not vary according to who obtained consent (research nurse versus others; *P* = 0.683), but did according to trial (*P* < 0.001).

**Table 3:** **Distribution of overall DelibeRATE scores** ^**a**^

Score	***n***(%)
≤10	0 (0)
11	1 (1)
12	1 (1)
13	1 (1)
14	2 (1)
15	1 (1)
16	5 (4)
17	6 (4)
18	15 (11)
19	18 (14)
20	82 (62)

^a^Five participants are not included in this table due to missing data on individual DelibeRATE items.

**Table 4:** **Response frequency across DelibeRATE items**

Item	Response	***n***(%)
1	Yes	134 (99)
	Unsure	2 (1)
	No	0 (0)
2	Yes	135 (99)
	Unsure	1 (1)
	No	0 (0)
3	Yes	126 (93)
	Unsure	10 (7)
	No	0 (0)
4^a^	Yes	114 (84)
	Unsure	16 (12)
	No	5 (4)
5	Yes	129 (95)
	Unsure	7 (5)
	No	0 (0)
6^b^	Yes	109 (81)
	Unsure	23 (17)
	No	2 (1)
7^a^	Yes	116 (86)
	Unsure	18 (13)
	No	1 (1)
8^a^	Yes	106 (79)
	Unsure	25 (19)
	No	4 (3)
9^a^	Yes	130 (96)
	Unsure	5 (4)
	No	0 (0)
10^b^	Yes	130 (97)
	Unsure	3 (2)
	No	1 (1)

^a^Item not completed by one participant. ^b^Item not completed by two participants.

**Table 5:** **DelibeRATE total score and time to decide by factor**

Variable	Factor	Median (IQR)	***P***-value
DelibeRATE total score	Educational attainment		0.528
	No formal education	20 (18 to 20)	
	Secondary	20 (18 to 20)	
	Higher	20 (20 to 20)	
DelibeRATE total score	Previous trial participation		0.859
	Yes	20 (19 to 20)	
	No	20 (18 to 20)	
Time to decide	Trial		<0.001
	Trial 1	8 (0.08 to 35)	
	Trial 2	0.4 (0.02 to 7)	
	Trial 3	0.125 (0.08 to 1)	
Time to decide	Research nurse taking consent		0.683
	Yes	1 (0.04 to 12)	
	No	0.125 (0.04 to 14)	

## Discussion

### Principal findings

This is the first study to prospectively measure the deliberation stage of decision-making for trial participation. Moreover, this study has included potential participants from a diverse range of trials across interventions, conditions and clinical areas (although the sample size is small), both those who subsequently consented to the respective trial and those who did not. There was no difference in overall DelibeRATE scores or between trial consenters and refusers. We identified additional potential problems with decision-making for trial participation from both the perspective of measurement of the process and more generally in relation to the overall trial.

The lack of a difference in DelibeRATE scores between trial consenters and refusers requires reflection. The most likely reasons for a lack of variability in the scores of consenters and refusers are deficiencies in the tool itself and the likelihood that the participants in this study are an unrepresentative sample of all potential recruits to the parent trials (reflected in the small numbers of refusers). Only 6% of those who completed the DelibeRATE questionnaire went on to refuse entry into the main trial, with 94% eventually consenting to participation. The lack of a statistically significant difference in DelibeRATE scores between consenters and refusers is not surprising, given the small number of refusers included in the sample. It is also plausible that a higher degree of variability between the two groups would be observed in a larger and more representative sample of refusers. The 94% consent rate is not a true representation of the consent rates in the parent trials, which had recruitment rates (adjusted for DelibeRATE sites) of 77% (trial 1), 72% (trial 2) and 68% (trial 3), giving a combined consent rate of 72%. There are several explanations for this misrepresentation from both a participant and a recruiter perspective.

From a participant perspective, it is likely that those individuals who are unconditional refusers (whether absolutely or temporally at this point in time) are unlikely to engage in any research study, whether it be a clinical trial or a less burdensome questionnaire-based study such as this one. The other group of refusers, and the more interesting one in relation to decision-making, are those who are of two minds about participation. It is unlikely that all of these ‘uncertains’ converted to trial consenters and, as such, more likely that this group of individuals was lost at an earlier stage of the study.

The other explanation for the sample in this study being overrepresented by trial consenters could be related to the recruiter. For all trial recruiters, there is a pressure to recruit maximum numbers of participants into the trial, and, as such, implicit judgements about the participants ‘fit’ for the trial may be made in advance of or during the discussion about trial participation. In other words, recruiters approach only potential participants who they think will say yes so as not to waste time on those who will refuse, ultimately improving recruitment efficiency but having detrimental consequences for the trial’s generalisability. There is evidence in the literature to support the notion that recruiters make ‘off-protocol’ decisions about potential participants. For example, researchers in one study reported recruiters’ targeting the ‘good study patient’, who were those perceived as ‘meticulous, proactive and compliant’ with good communication skills and strong social networks to support their participation [19]. In a recent study, Donovan and colleagues found that both doctors and nurses struggle with their role as ‘recruiters’ and make implicit judgements about patients’ eligibility largely based on a lack of equipoise [20]. Research nurses also report using nonverbal cues from potential participants as a way to evaluate whether these individuals are willing to participate (K Gillies, personal communication). In addition, recruiters may perceive DelibeRATE as a measure of their ability to inform potential participants, even though they were assured this was not the aim of the study. As such, they may have changed their behaviour to select only appropriate or ‘good’ patients or may have spent more time discussing aspects of the trial than would be standard practice. However, it is unlikely that all recruiters across the seven included sites attempted to elicit this behaviour and, as such, would not be accountable for the entire 94% consent rate.

With regard to the design of the study, it is likely that, for a large proportion of participants included in this study, the decision-making measurement was taken after a commitment to the decision had already been made; that is, the DelibeRATE questionnaire was measuring a decision that had already been made rather than the deliberative process, which had happened earlier. This speculation that the measurement was postcommitment is further supported by the high overall scores seen in the sample. These high scores may also be supportive of the recruiters’ approaching only the ‘good’ participants, but data on the number of questionnaires distributed versus those returned would be required for this finding to be more conclusive. Unfortunately, we did not collect data on the specific response rate regarding participation to this study. However, a very rough estimate of 87% for the response rate can be made on the basis of the number of questionnaires actually distributed at sites (based on data from three of the seven sites). This estimate, along with the adjusted recruitment rate for each parent trial, suggests that the proportion of trial refusers was likely higher than 6% but that those participants were not amenable to completing the DelibeRATE questionnaire. The final consideration regarding the influence of study design on the results is the development of the DelibeRATE tool. The tool was developed for use in a treatment context, and, though the constructs being measured (that is, those items related to the steps of deliberation) would be the same irrespective of the index decision, the framing of the constructs was changed without assessment of their validity before use. Therefore, any lack of difference in scores may also reflect deficiencies in the tool in this context.

It is worth discussing in general how scores generated from measures of informed consent (both outcomes and process) would be operationalised in this context. Whereas other measures of informed consent highlight a deficit in outcomes postdecision, when it may be too late to intervene, we measured aspects of the decision-making ‘process’ for trial participation prospectively and, as such, our study had the potential to detect ‘poor’ decisions as they were being made. Exactly how this or similar measures would be operationalised requires more thought. For example, if the maximum score is 20, is there a requirement for all participants to achieve 100% on their decision-making process measure before their subsequent consent is considered valid? Or is there a threshold at which consent becomes acceptable, and is this threshold based on the items’ having equal weighting or are some more important than others? In this study, 95% of the participants scored ≥17 (85%), but does that mean that the 5% who scored ≤16 (80%) gave suboptimal consent, that is, that their decision process was not as good as it could have been, and, as such, that their consent should be questioned? Maybe a better assessment method would be to follow up these participants over time and investigate scores in relation to retention in the trial. It may be that retention is in fact a better measure of decision quality than recruitment and thus should be explored in future design and analysis of measures of decision-making about trial participation.

### Strengths and limitations

The two main limitations of this study are (1) that the study sample was composed predominantly of those who subsequently consented to trial participation and (2) the use of a tool to measure decision-making that had not been designed and specifically validated for use in a trial consent context. The tool was not formally piloted, but was tested for comprehension amongst colleagues. Therefore, the lack of variability in the DelibeRATE scores could be due to a lack of validity and reliability of the measurement instrument itself. Further work required to validate this tool could include (but is not limited to) pilot testing with potential participants to assess face validity and explore item phrasing and redundancy (using cognitive interviews) to investigate content validity. Assessment of reliability, specifically internal consistency, could be conducted using Cronbach’s α analysis of linked items and test–retest reliability to evaluate the stability of the measure over time. The primary strength of this study is that it has demonstrated that it is possible to prospectively measure potential participants’ decision-making about trial participation, albeit in a small and selective sample underrepresenting those who are more likely to refuse trial participation. Measuring aspects related to informed consent (including decision-making) in a fully representative population of potential trial participants is very difficult. Most studies in which investigators have assessed aspects of the trial participation decision, described earlier, have been conducted in cancer trials with decisions measured retrospectively, which may be biased by outcomes attributed to the decision or at best immediately following the decision (that is, consent) [10]. A further strength of this study is that we investigated decision-making across a range of conditions, clinical areas, geographic sites and recruiters. Additionally, the nonstandard conceptualisation and operationalisation of the informed consent process for clinical trials applied in this study is a further strength. Specifically, in this study, we approached informed consent as a decision-making process and measured constructs associated with a stage of that process rather than focusing on outcomes such as understanding. A comparison of the DelibeRATE tool and commonly used alterative measures of informed consent is presented in Table 6. This table highlights the difference in conceptualisation and timing of the DelibeRATE tool compared to existing trial consent measures.

**Table 6:** **Comparison of DelibeRATE tool with existing measures of informed consent** ^**a**^

Instrument	Year	Population	Theoretical/conceptual framework	Constructs assessed	Items	Timing	Sample questions
DelibeRATE	2012	Set within three different parent RCTs: two surgical and one drug, all phase III pragmatic RCTs of direct head-to-head comparisons	Structured around a conceptual framework informed by theories of decision-making which separates decision-making process into deliberation and determination	1. Information search	10 items	Measured before consent to participate in trial	I understand the option, of participating in the trial or not, is available to me
				2. Knowledge gain			
				3. Appraisal of knowledge sufficiency			
				4. Imagining counterfactuals			
							I know how I feel about participating in the trial or not
				5. Affective forecasting			
				6. Preference construction			
Quality of Informed Consent (QuIC) [6]	2001	Patients and parents of paediatric patients enrolled in phase I, II or III clinical trial; tool developed with intention to be used across clinical areas	Conceptual framework considered: existing theoretical work on therapeutic misconception, regulations governing research, recommendations of National Cancer Institute working group	1. Objective understanding (part A)	Total = 34 items	After consent	I have been informed how long my participation in this clinical trial is likely to last (part A)
					Part A: Objective understanding (*n* = 20)		
				2. Subjective understanding (part B)			
				Based on 13 domains identified in regulatory documents on informed consent	Part B: Subjective understanding (*n* = 14)		
							The fact that your treatment involves research (part B)
Informed Consent Assessment Instrument (ICAI) [9]	2013	Set within an open-drug RCT for tuberculosis	No explicit theory reported	Content informed by the four principles of research ethics: autonomy, beneficence nonmaleficence and justice	Total = 10 items	Measured right after time of consent, then remeasured 8 to 10 weeks after consent	Are you participating in a clinical trial?
			Authors state informed by principles of research ethics				
							Are the risks and benefits of taking part in the study clear?
				Format informed by QuIC			
Brief Informed Consent Evaluation protocol (BICEP) [10]	2005	Set within eight different parent RCTs	No explicit theory reported; conceptual dimensions: therapeutic misunderstanding, voluntariness and understanding	Autonomous authorisation	Total = 15 items	Measured immediately after consent process	What is the primary purpose of [*parent study*]?
					Informed consent aggregate score (ICAS) (*n* = 10)		
							What are the benefits to you of participating in the [*parent study*]?
					Therapeutic misconception aggregate score (TMAS) (*n* = 5)		

^a^RCT, Randomised controlled trial.

### Key recommendations

Decisions about whether to participate in a clinical trial are suboptimal [4, 5]. This is not surprising when one considers that items important for ‘good’ decision-making are lacking in existing patient information leaflets for clinical trials [21]. Designers of interventions to improve the decision-making process have tended to address understanding by focusing on the content and structure of provided information [4, 5]. The results of some studies have highlighted the need for new ways of thinking about assessing the decision-making process for trial participation. Miller *et al*. concluded that a measure of preparation for trial participation decision-making is needed [8]. In line with this, interventions that recognise the decision and what it means for participants are being developed [22–25]. As these interventions are developed, appropriate measures to evaluate their effectiveness, which go beyond understanding, are also required.

More work needs to be done with regard to how to access the trial refusers so as to measure and explore their decision-making in more depth to ensure both consenters and refusers are making decisions in line with their personal values and preferences. It may be that studies aimed at measuring decision-making in this context need to be more embedded in the parent trial, such that they become invisible and seamless within the trial. Appropriate ways to engage potential participants with methods to capture decision-making data should be explored. For example, using novel methods to capture data (such as with tablet computers) could have potential. Moreover, involving patients and the public in designing studies of this type could improve the development of the tool and its implementation for data capture. Further work is also required to develop a tool that accurately captures the conceptual aspects of decisions about trial participation, being mindful of both the regulatory framework of informed consent and the goals and values of the individual. These tools should adopt a ‘fast and frugal’ approach to their measurement, in terms of both the underlying theoretical framework and their design and delivery [26].

## Conclusions

Our study shows that decisions about trial participation can be measured prospectively during the recruitment process in clinical trials in different contexts. However, further work is required to determine whether the lack of variability in the DelibeRATE scores in our study (both at the level of the entire sample and between consenters and refusers) is due to the nonvalidated nature of the tool or to the underrepresentation of trial refusers in the study sample. We highlight points important to consider by researchers designing trials and those involved in the informed consent process, such as recognition that potential participants may have made a decision about ‘clinical trials’ even before they were invited to participate and that recruiters may make implicit judgements about potential participants during the trial recruitment process. We propose considerations for future studies designed to measure decision-making in this context and studies embedded within the recruitment process for clinical trials.

## Abbreviations

CHaRT: Centre for Healthcare Randomised Trials
QuIC: Quality of Informed Consent.
